# Fall-related emergency department visits and hospitalizations among community-dwelling older adults: examination of health problems and injury characteristics

**DOI:** 10.1186/s12877-019-1329-2

**Published:** 2019-11-11

**Authors:** Namkee G. Choi, Bryan Y. Choi, Diana M. DiNitto, C. Nathan Marti, Mark E. Kunik

**Affiliations:** 1 The University of Texas at Austin Steve Hicks School of Social Work, 1925 San Jacinto Blvd, Austin, TX 78712 USA; 2 Department of Emergency Medicine, Brown University Warren Alpert Medical School, Providence, RI USA; 3 Houston VA HSR&D Center for Innovations in Quality, Effectiveness and Safety, Michael E. Debakey VA Medical Center; VA South Central Mental Illness Research, Education and Clinical Center; and Baylor College of Medicine, Houston, TX USA

**Keywords:** Falls, Fall injury, ED, Hospitalization

## Abstract

**Background:**

Fall injuries and related healthcare use among older adults are increasing in the United States. This study examined chronic illnesses, sensory and memory problems, and injury characteristics that were associated with ED visits and hospitalizations among older adults who received medical attention for fall injuries within a 91-day reference period.

**Methods:**

Data were from the publicly available 2013–2017 US National Health Interview Survey files (unweighted *N* = 1840 respondents aged > 60 years with fall injuries). We first described socioeconomic, health/mental health, healthcare utilization, and injury characteristics among three groups: those who neither visited an ED nor were hospitalized for their fall injury, those who visited an ED only, and those who were hospitalized. Then, using multinomial logistic regression analysis, we examined associations of healthcare utilization (ED visit only and hospitalization vs. no ED visit/hospitalization) with chronic illnesses, other health problems, and injury characteristics, controlling for socioeconomic factors.

**Results:**

Of older adults who received medical attention for fall injuries, a little more than one-third had an ED visit only and a little less than a fifth had an overnight hospital stay. Multivariable analysis showed that lung disease and memory problems were associated with higher risk of ED visit only; hip and head injuries, facial injuries, and broken bones/fractures (from any type of injury) were more likely to result in hospitalization than other injuries. Fall injuries sustained inside the home, falls from loss of balance/dizziness, and living alone were also more likely to result in hospitalization.

**Conclusions:**

These healthcare utilization findings indicate the significant toll that fall injuries exact on older adults and healthcare systems. Fall prevention should target risk factors that are specific to serious injuries requiring costly care. Strategies for implementing scalable, adaptable, and measurable fall prevention models by primary care and emergency medical service providers and ED staff are needed.

## Background

Falls, though preventable, are common among older adults, and the resulting injuries can threaten their health, independence, and lives. In 2014, 30% of older adults (aged > 65 years) in the United States reported falling at least once, and the estimated 29.0 million falls that year resulted in 7.0 million injuries and approximately 27,000 deaths [1]. Fall injuries often require costly medical intervention. In 2014, 2.8 million US older adults were treated in emergency departments (ED) for fall-related injuries, and approximately 800,000 of them were subsequently hospitalized, most often due to a head injury or hip fracture, at an average cost of over $30,000 per hospitalization [1–3]. The total estimated medical cost attributable to fatal and nonfatal falls was approximately $50.0 billion in 2015, including fees for hospital and nursing home care, doctors and other professional services, rehabilitation, community-based services, use of medical equipment, prescription drugs, and insurance processing [4].

As Americans age, falls, fall injuries and deaths, and fall-related healthcare usage are all projected to rise. Between 2003 and 2010, the ED visit rate for falls and fall injuries among those aged ≥65 increased from 60.4 to 68.8 per 1000 population, with the largest increase among those aged 75–84 and higher rates among women than men [5]. The Centers for Disease Control and Prevention (CDC) report also showed that between 2007 and 2016, age-adjusted fall death rates among older adults increased 31%, with the largest increase per year among those aged ≥85 and higher rates among men than women [6].

Despite increases in fall injuries and related healthcare use, we found no studies that have examined health/mental health and fall injury characteristics associated with ED visits or hospitalizations. To improve targeting of fall prevention efforts, we examined associations between health/mental health and fall injury (site, location, and cause) characteristics and ED visits and hospitalizations among community-dwelling older adults within a 91-day reference period. We included those aged ≥60, because fall injury rates among those aged 60–64 are similar to those among people aged ≥65 [7]. Our exploratory hypothesis was that compared to those who did not utilize ED or inpatient hospital services for their fall injury, those who had an ED visit without hospitalization or who were hospitalized would have more chronic illnesses and sensory and memory problems, be more likely to have sustained hip, head, and face and fracture injuries, and would be more likely to be injured at home due to loss of balance or dizzy spells, controlling for socioeconomic factors. Given the lack of previous research, we refrained from hypothesizing about differences in health/mental health and injury characteristics between those who had an ED visit only versus a hospitalization with or without an ED visit.

## Methods

### Data and sample

We utilized the 2013–2017 public use data files of the National Health Interview Survey (NHIS), an annual, cross-sectional household survey which is the principal source of information on the health and healthcare access of the civilian, noninstitutionalized US population [8]. For each sampled household, interviews are conducted (mostly face-to-face) with an adult family member who answers questions about the demographic and health status characteristics of each family member. For this study, we linked data on medically attended injury/poisoning episodes that occurred to any family member within the 91-day reference period to their demographic and other health data. Combining all 5 years of NHIS data resulted in a sample of 495,663 individuals aged < 1 to 85+ years (NHIS public use data sets do not provide chronological age of those aged > 85 years). Of these, 104,340 were aged > 60, and of them, 1800 had other injuries (e.g., due to motor vehicle or other mobility means, cutting/piercing, burns, poisoning) than falls, and 1840 (representing 1.16 million individuals over the 5-year study period) had fall injuries. We focused on those with fall injuries to address study questions. In the case of those with more than one fall injury episode during the 91-day period, the most recent episode was used for analysis.

### Measures

#### Healthcare utilization

For each injury episode, questions were asked about whether or not injury care was received through a call to a medical professional, at a doctor’s office or clinic, at an ED, and/or at any place else. Questions were also asked about whether the person was hospitalized and the number of nights hospitalized. Responses to these questions led to identifying the following three groups of fall victims: those without ED visit or hospitalization (reference group), those who had an ED visit only, and those who were hospitalized.

#### Diagnosed chronic illnesses and sensory and memory problems that cause limitations

Diagnosed chronic illnesses that caused limitations included arthritis, cancer, diabetes, high blood pressure, heart disease, lung cancer, and stroke. We also included vision, hearing, and memory problems that caused limitation as possible correlates of fall injuries.

#### Other health conditions

For descriptive purposes only, we present number of activities of daily living (ADL) impairments (0–6; feeding, bathing, getting dressed, toileting, transferring to/from bed or chairs, and getting around in the home), difficulty walking without equipment, and limitations caused by depression/anxiety (yes = 1, no = 0 for each). Past-year healthcare use is also presented to describe the sample. All of these may have been affected by fall or other injuries among the injured individuals.

#### Fall injury site and broken bones or fractures

For each fall (or other) injury episode, respondents were asked to list up to four parts of their body hurt due to the injury and “how” each body part was affected. We collapsed the answers regarding fall injury sites into eight categories (e.g., hip, head, face, lower and upper limbs). Broken bones/fractures were distinguished from all other types of fall injury (e.g., sprains, cuts, scrapes, bruises, burns).

#### Fall location

Response categories for location were at the injured person’s home or outside (sidewalk, parking lot, sports facility, shopping mall, and so forth). Because most older adults’ falls occurred at home, we categorized the responses into (1) inside the home, (2) at home but outside (e.g., yard, patio), and (3) away from home. Questions were also asked about whether the fall involved the floor/level ground, stairs/steps, bathtub/shower toilet, ladder/scaffolding, sports field/court/rink, and so forth. As these categories are highly correlated with fall location, we present them for descriptive purposes only.

#### Cause of fall

The response categories were slipping/tripping, loss of balance or dizziness, bumping into an object or another person, being shoved or pushed by another person, jumping or diving, or other. For parsimony, we categorized them into slipping/tripping, loss of balance or dizziness, and other.

#### Socioeconomic variables

These included age (60–69; 70–79; and 80+ years); gender, race/ethnicity; marital status, living arrangement (alone vs. with someone), education (college degree vs. no college degree), family income to poverty ratio, and health insurance types (Medicare, Medicaid, Veterans Administration and other insurance for military personnel, and/or private health insurance).

### Data analysis

All analyses were conducted with Stata/MP 15’s svy function to account for NHIS’ stratified, multistage sampling design. First, to describe the sample, we used χ^2^ and *one-way ANOVA* tests to compare socioeconomic, clinical, and healthcare utilization characteristics of the three groups of older fall victims by their healthcare utilization (no ED visit or hospitalization, ED visit only, and hospitalization). We also used χ^2^ tests to compare injury site, fracture, location, and cause among the three groups. We did not adjust reported *p* values due to a number of considerations [9]; however, it is important to acknowledge that 5% of tests represent a Type I error. To test H1 (correlates of ED visit only and hospitalization vs. no ED visit or hospitalization), we used multinomial logistic regression analysis. To identify a parsimonious model, we used backward elimination and excluded the following nonsignificant socioeconomic factors: race/ethnicity, marital status, past-year work status, family income to poverty ratio, and health insurance type. Survey years (2013 vs. 2014, 2015, 2016, and 2017) were also excluded from the final model because they were nonsignificant as a covariate of healthcare utilization. Variance inflation factor diagnostics, using a cut-off of 2.50 [10], showed that multicollinearity among the included covariates was not a concern. Results of the multinomial logistic regression model are presented as relative risk ratios (RRR) with 95% confidence intervals (CI). Statistical significance was set at *p <* .05.

## Results

### Characteristics of fall victims by type of healthcare use

Table 1 shows that nearly a third of all fall victims were aged > 80; nearly a third was widowed; nearly a third had family income <2x the federal poverty threshold; and 44.4% had difficulty walking without equipment. Of all fall victims, 46.9% neither went to an ED or were hospitalized (no ED/hospitalization group), 33.7% had an ED visit only (ED-only group), and 19.4% were hospitalized (hospitalized group). Of these three groups, the hospitalized group (who spent an average of 11.2 nights [SE = 1.32] in hospitals) included the highest proportion of the 80+ age group and had the highest rate of prior stroke and the highest rates of ADL and mobility impairments. Compared to the no ED/hospitalization group, both the ED-only and hospitalized groups were less likely to have a college degree and higher income, but were more likely to live alone and have Medicare and a higher rate of memory problems. The three groups did not differ on the prevalence of six chronic illnesses, vision and hearing problems, depression, and anxiety.

**Table 1:** Characteristics of older adults with fall injury by emergency department (ED) visit and/or hospitalization status

	All 1840 (100%)	No ED visit or hospitalization 875 (46.85%)	ED visit only 612 (33.71%)	Hospitalization 353 (19.44%)	Oveall *p*	ED only vs. hosp. *p*
Age group (yrs, %)					<.001	<.001
60–69	38.28	45.03	36.36	25.35		
70–79	30.02	29.19	29.36	33.14		
80+	31.70	25.78	34.28	41.51		
Female (%)	65.59	64.87	68.02	63.08	.389	.204
Race/ethnicity (%)					.296	.726
Non-Hispanic White	82.11	80.90	82.76	83.89		
Non-Hispanic Black	7.69	7.80	7.59	7.58		
Hispanic	6.83	6.73	7.58	5.77		
Other	3.38	4.57	2.07	2.76		
Marital status (%)					.005	.337
Married/cohabiting	46.66	52.21	43.59	38.57		
Widowed	30.25	25.85	31.66	38.42		
Divorced/separated	17.31	16.45	18.19	17.87		
Never married	5.78	5.49	6.56	5.14		
Living alone	38.83	35.71	41.23	42.19	<.001	.806
College degree	27.86	31.47	25.98	22.42	.012	.292
Worked for pay last year (%)	18.69	24.59	15.51	10.00	<.001	.035
Family income to poverty ratio (%)					.007	.009
Under 2x poverty	32.67	30.29	31.09	41.17		
2–3.99x poverty	26.03	27.01	24.59	26.11		
4 + x poverty	34.17	31.08	29.16	22.01		
Missing	15.30	11.68	15.16	10.71		
Health insurance (%)						
Medicare	81.25	78.17	82.03	87.23	.005	.065
Medicaid	12.46	13.91	10.10	13.07	.157	.222
VA/military insurance	9.46	9.33	10.51	7.97	.503	.254
Private health insurance	50.70	53.47	50.88	43.72	.031	.071
Number of chronic illnesses that caused limitations (M. SE)	0.73 (0.03)	0.75 (0.05)	0.69 (0.05)	0.77 (0.02)	.546	.250
Arthritis (%)	21.42	21.81	21.96	19.54	.729	.472
Cancer (%)	4.43	4.90	3.81	4.37	.708	.727
Diabetes (%)	10.45	10.54	9.58	11.71	.690	.403
High blood pressure (%)	11.71	13.91	9.08	10.93	.054	.426
Heart disease (%)	12.51	12.14	11.46	15.22	.295	.125
Lung disease (%)	7.58	6.51	8.61	8.37	.337	.906
Stroke (%)	5.17	5.04	4.14	7.29	.146	.041
Vision problem (%)	7.42	8.25	7.37	5.52	.376	.361
Hearing problem (%)	5.80	5.88	5.30	6.47	.817	.549
Memory problem (%)	19.91	17.32	20.17	25.72	.019	.091
Number of ADL impairments (M,SE)	0.68 (0.04)	0.48 (0.05)	0.65 (0.07)	1.21 (0.11)	<.001	<.001
Difficulty walking w/o equipment (%)	44.36	36.63	43.92	63.79	<.001	<.001
Depression/anxiety (%)	7.08	6.80	7.23	7.47	.926	.903
Past-year healthcare utilization (%)						
Hospitalization	36.01	22.26	22.21	93.08	<.001	<.001
Homecare	16.11	11.0	12.80	34.17	<.001	<.001
10+ times healthcare use	38.64	37.61	35.29	46.93	.008	.004
Healthcare for fall injury (%)						
Call to medical professional	36.92	38.18	31.89	42.60	<.001	.006
Doctor’s office/clinic	72.50	87.16	60.07	58.95	<.001	.735
Emergency medical service (ambulance, fire truck) use	31.46	11.19	37.57	69.70	<.001	<.001
Emergency department visit	51.82	0	100	93.14	<.001	<.001
Hospitalization	19.44	0	0	100		
Other place	14.32	15.64	8.19	21.76	<.001	<.001

With respect to healthcare use for fall injuries, nearly three-quarters visited a doctor’s office/clinic, more than half went to an ED, more than a third called medical professionals, and nearly a third used emergency medical service (EMS; presumably for a “lift assist” to get off the floor and/or for ED or hospital transport). A little less than a fifth had an overnight hospital stay. The no ED/hospitalization group had the highest rate (87.2%) of visits to a doctor’s office/clinic. The hospitalized group had the highest incidents of past-year hospitalization and homecare use, and overall, the most frequent healthcare use (noted by using healthcare 10+ times in the preceding year). The hospitalized group also had the highest rates of calls to medical professionals, EMS use, and care in other places for fall injuries, and 93.1% were admitted via the ED.

### Fall injury site, fractures, and location and cause of injury by type of healthcare use

Table 2 shows that of all fall victims, injuries to lower limb (32.1%), upper limb (23.1%), shoulder, neck, back, and/or buttocks (23.9%) were most common, followed by injuries to head (15.3%), hip (11.1%), and face, ear, jaw, and/or teeth (10.6%), and 30.9% had one or more broken bones or fractures. Of the three groups, the no ED/hospitalization group had a higher rate of lower limb injuries (40.7%) than the other two groups. Compared to the no ED/hospitalization group, both the hospitalized and ED-only group had higher rates of head injuries (19.1 and 22.1% vs. 8.7%, respectively) and face, ear, jaw, and/or teeth injuries (10.6 and 14.1% vs. 8.1%, respectively). The hospitalized group had the highest rates of hip injury (24.6%) and broken bones/fractures (53.4%). Additional analysis showed that the average number of nights hip injury patients spent in a hospital (11.8 [SE = 1.85]) did not differ significantly from all other hospitalized fall injury patients (10.9 [SE = 1.66]) (*p* = .730).

**Table 2:** Fall injury site, fracture, and location and cause of injury

	All 1840 (100%)	No ED visit or hospitalization 875 (46.85%)	ED visit only 612 (33.71%)	Hospitalization 353 (19.44%)	Overall *p*	ED only vs. hosp. *p*
Injury site (%)						
Leg, knee, ankle, foot, and/or other parts of lower limb	32.06	40.65	26.39	21.18	<.001	.103
Arm, elbow, hand, and/or other parts of upper limb	23.12	24.76	23.40	18.68	.155	.138
Shoulder, neck, back, and/or buttocks	23.90	25.77	21.92	22.83	.309	.778
Hip	11.06	9.94	4.82	24.59	<.001	<.001
Head	15.26	8.74	22.13	19.07	<.001	.327
Face, ear, jaw, teeth	10.57	8.05	14.07	10.57	.011	.207
Chest, stomach, and/or groin	3.82	4.27	4.20	2.11	.253	.089
Other	12.07	10.69	13.33	13.23	.331	.970
Any broken bone or fracture (%)	30.87	18.11	35.62	53.37	<.001	<.001
Fall location (%)					<.001	.005
Home (inside)	51.48	46.87	50.82	63.76		
Home (outside)	23.76	28.47	21.73	15.90		
Away from home	24.76	24.66	27.45	20.34		
How the person fell (%)					.584	.788
Floor/level ground	39.95	38.38	40.08	43.54		
Stairs/steps/escalator	13.78	14.15	13.13	14.03		
Bed/chair/sofa	7.36	7.09	8.17	6.60		
Curb/sidewalk	7.26	9.02	6.20	4.89		
Bathtub/shower/toilet	3.89	3.63	3.73	4.78		
Other	27.76	27.74	28.70	26.17		
Cause of fall (%)					<.001	<.001
Slipping/tripping	59.04	57.41	54.96	34.04		
Loss of balance/dizziness	24.42	20.27	22.57	37.64		
Other	23.53	22.31	22.47	28.31		

More than one-half of falls occurred inside the person’s home, almost a quarter also occurred at home but outside, and the remaining quarter occurred away from home. Almost 40% involved falling on the floor/level ground, and nearly 14% occurred on stairs/steps/escalators. Almost 60% of falls were caused by slipping or tripping over some objects and nearly a quarter by loss of balance or dizziness. The hospitalized group was most likely to have fallen inside their home due to loss of balance or dizziness.

### Correlates of ED use only and hospitalization versus no ED visit/hospitalization: multinomial regression analysis results

Table 3 shows that those with high blood pressure and lower limb injuries were less likely to use an ED or to be hospitalized for their injury, while those with upper limb, chest, stomach, and groin injuries were less likely to be hospitalized. Those with lung disease and memory problems were more likely to use the ED only than to be hospitalized. Those with face, ear, jaw, teeth, and head injuries were more likely to use the ED and to be hospitalized. Those with a hip injury were less likely to use the ED only (RRR = 0.47, 95% CI = 0.28–0.79) but more likely to be hospitalized (RRR = 3.05, 95% CI = 1.93–4.80). Having a broken bone or fracture was associated with a higher likelihood of ED use only (RRR = 3.59, 95% CI = 2.58–5.00) and hospitalization (RRR = 9.50, 95% CI = 6.43–14.04).

**Table 3:** Association of ED visit and hospitalization with health/mental health and fall injury characteristics

	No ED visit or hospitalization vs.	
	ED visit only RRR (95% CI)	Hospitalization RRR (95% CI)
Arthritis (vs. no arthritis)	1.27 (0.88–1.83)	0.98 (0.61–1.59)
Cancer (vs. no cancer)	0.78 (0.40–1.52)	0.67 (0.25–1.81)
Diabetes (vs. no diabetes)	0.98 (0.58–1.66)	1.24 (0.72–2.15)
High blood pressure (vs. no high blood pressure)	0.46 (0.26–0.79)**	0.44 (0.23–0.84)*
Heart disease (vs. no heart disease)	0.98 (0.61–1.58)	1.22 (0.68–2.17)
Lung disease (vs. no lung disease)	2.04 (1.15–3.60)*	1.57 (0.82–3.01)
Stroke (vs. no stroke)	0.78 (0.42–1.45)	1.18 (0.56–2.46)
Vision problem (vs. no vision problem)	0.86 (0.51–1.44)	0.48 (0.18–1.27)
Hearing problem (vs. no hearing problem)	0.93 (0.50–1.72)	1.56 (0.63–3.85)
Memory problem (vs. no memory problem)	1.45 (1.00–2.09)*	1.53 (0.99–2.37)
Injury site (vs. no injury at site)		
Leg, knee, ankle, foot, and/or other parts of lower limb	0.55 (0.40–0.77)***	0.63 (0.42–0.94)*
Arm, elbow, hand, and/or other parts of upper limb	0.73 (0.53–1.00)	0.61 (0.39–0.94)*
Chest, stomach, and/or groin	0.68 (0.34–1.36)	0.25 (0.10–0.63)**
Shoulder, neck, back, and/or buttocks	0.84 (0.59–1.19)	1.03 (0.70–1.50)
Face, ear, jaw, teeth	2.11 (1.32–3.38)**	2.60 (1.38–4.90)**
Hip	0.47 (0.28–0.79)**	3.05 (1.93–4.80)***
Head	3.45 (2.31–5.15)***	4.29 (2.51–7.34)***
Any broken bone or fracture (vs. none)	3.59 (2.58–5.00)***	9.50 (6.43–14.03)***
Fall location: Home (outside) vs.		
Home (inside)	1.31 (0.95–1.81)	1.98 (1.25–3.14)**
Away from home	1.59 (1.12–2.27)*	1.58 (0.96–2.60)
Cause of fall: Slipping/tripping vs.		
Loss of balance/dizziness	1.00 (0.72–1.39)	2.68 (1.81–3.99)***
Other	0.88 (0.63–1.22)	1.75 (1.15–2.65)**
Age group: 60–69 years vs.		
70–79	1.10 (0.80–1.52)	1.56 (1.04–2.33)*
80+	1.27 (0.92–1.76)	1.49 (0.99–2.26)
Female (vs. male)	1.02 (0.76–1.38)	0.70 (0.48–1.01)
College or higher degree (vs. no college degree)	0.72 (0.54–0.95)*	0.59 (0.40–0.85)*
Living alone (vs. not living alone)	1.29 (0.98–1.70)	1.57 (1.09–2.27)*
*N* = 1840; design df = 869; *F* (54,816) = 7.05; *p* < .001		

*p < .05; **p < .01; ***p < .001

Those who sustained a fall injury away from home had a higher likelihood of ED use only (RRR = 1.59, 95% CI = 1.12–2.27), while those who sustained a fall injury inside their home were more likely to be hospitalized (RRR = 1.98, 95% CI = 1.25–3.14). Fall cause was not associated with the likelihood of ED use only, but both loss of balance/dizziness (RRR = 2.68, 95% CI = 1.81–3.99) and other causes (RRR = 1.75, 95% CI = 1.15–2.65), as opposed to slipping/tripping, were associated with a higher likelihood of hospitalization.

Of the control variables, those 70–79 years of age (as opposed to 60–69 years of age) and living alone had a higher risk of hospitalization, while those with a college degree had a lower risk of both ED only and hospitalization, compared to those with no ED/hospitalization.

## Discussion

This study of correlates of healthcare use for fall injuries confirms the significant toll that fall injuries exact on older adults and healthcare systems. Though most chronic illnesses were not associated with older adults’ healthcare use for fall injuries, lung disease and memory problems were associated with higher risk of ED use for fall injuries. A fall victim with a chronic lung disease may report acute or chronic shortness of breath, prompting an ED evaluation, but may not require inpatient management. Memory impairment in older adults can also cause uncertainty about fall mechanisms, leading to a lower threshold for transport to the ED by family or EMS, but not necessarily for hospital admission. These findings mostly support our exploratory hypothesis about factors associated with ED and hospital use for fall-related injuries. The negative association between ED or hospital use and high blood pressure requires more research. Patients with hypertension may be more likely to see their primary care providers frequently and to also rely on them for post-fall medical attention.

Our findings about fall injuries, fall locations (mostly at home), causes of falls (loss of balance or a slip/trip) are congruent with the findings from 371 falls during a four-year period from 120 fallers [11]. However, our study also expands knowledge about other fall injury characteristics that contribute to different types of healthcare use. Hip and head injuries, facial injuries, and broken bones/fractures (from any type of injury) were more likely to require hospitalization than injuries to other parts of the body. The finding that older adults with hip injuries were less likely to use the ED only and more likely to be hospitalized is likely because many hip injuries seen at the ED required subsequent hospitalization. By comparison, those with head and facial injuries were equally likely to use the ED only and to be hospitalized, suggesting that these injuries are less likely than hip injuries to require hospitalization after an ED visit. Fall injuries sustained inside the home were more likely to result in hospitalization, while those that occurred away from home were more likely to result in ED use only. Falls from loss of balance/dizziness were more of a risk factor for hospitalization than slipping and tripping, presumably because loss of balance/dizziness may be correlated with greater underlying health problems and frailty, while slipping and tripping episodes are more preventable.

Fall victims aged 70–79 years (compared to those aged 60–69 years) and living alone were also more likely to be hospitalized. ED providers may have been uncomfortable discharging them back to their homes if they lacked available caregivers or due to inability to quickly arrange for home health services. Those with a college degree were less likely to use an ED or be hospitalized, perhaps because they may have had had greater access to other care resources following fall injury. A recent study of older Medicare beneficiaries in the US found that fall-related injuries overall ranked as the third-leading readmission diagnosis within 30 days of hospital discharge and accounted for 5.1% of all readmission dagnoses [12]. Among those with a fall injury and cognitive impairment at index admission, fall injuries were the second-leading diagnosis for readmission; and for those with a fall injury at index admission and discharged home or to home health care, fall injuries were the leading readmission diagnosis [12]. This shows that many older adults who are hospitalized for their fall injuries do not recover well and are likely to experience other health crises including recurrent falls.

Based on study findings, we recommend the following to reduce falls, fall injuries, and costly medical care. First, access to and effectiveness of fall prevention programs for older adults should be improved, especially for those who live alone and are at risk of falling at home due to balance problems, dizziness, and/or a slip/trip. While the evidence for group- and home-based exercise and multifactorial assessment and interventions for reducing falls is encouraging [13, 14], older adults who have balance/mobility problems and live alone may have limited access to community-based group exercise or other fall prevention programs. These older adults should receive in-home fall risk assessments, prevention education, and related interventions.

Second, for those at high risk of fall-related hip and head injuries, an assessment tool that can independently predict these risks should be used [15], and more effective fall prevention measures targeted at reducing these most serious and costly injuries are needed. In addition to treating underlying causes of falls, specific everyday strategies and behavioral modifications to improve mobility and stability and reduce these fall-related injuries should be disseminated to older adults, their family members, and other caregivers. Studies have shown relatively simple home-safety assessment and improvement and home-modifications are cost-effective fall injury prevention interventions [16–18]. Home modifications involving an occupational therapist have also been identified as having the potential to help the greatest number of older adults [19]. As most falls occur at home due to loss of balance and slipping and tripping, home modification and safe ambulation training are likely to be effective strategies.

Third, given our findings that nearly three quarters of older adults with fall injuries sought treatment at a doctor’s office/clinic, falls risk assessment and prevention strategies should be incorporated in primary care settings. While many geriatric specialists are already using these strategies, improving multifactorial falls risk assessment and management of older adults at high fall risk is needed in all primary care settings [20, 21]. A recent survey of primary care physicians found that nearly all of them believed that all older adults should be assessed for fall risk; however, only 52% believed that they had the expertise to conduct fall risk assessment [22].

Fourth, EMS providers who are called to older adults’ homes to provide lift assists are in a prime position to assess further fall risk and provide education and brief interventions to prevent these older adults from falling again [23]. The ED is also a logical place for multifactorial fall risk assessment and brief interventions for older adults with fall injuries. However, a recent study showed that most first responders offer no significant intervention or follow-up to prevent subsequent falls, even though some older adults place multiple fall-related calls to EMS over short time periods [24]. A systemic review also found little evidence of ED-based screening to prevent the need for future fall-related calls among older adults [25]. Implementation of scalable, adaptable, and measurable falls risk assessment and prevention models that are specified in geriatric emergency department guidelines are needed [26–28].

Fifth, hospitalization is inevitable when older adults have serious fall-related injuries like fractures. Hospital stays, especially those involving surgeries, are, however, likely to contribute to further deterioration of overall health and mental health, leading to discharge with home healthcare or to institutional settings [29]. As discussed, fall injuries are a leading cause of hospital readmission especially when older adults have cognitive impairment and are discharged home or to home healthecare [12]. Since a history of falls is also a strong predictor of recurrent falls [30, 31], post-discharge fall prevention interventions are essential. All healthcare and social service providers who serve older adults at risk of falls and recurrent falls should be trained in multifactorial and evidence-based fall risk assessment and falls prevention interventions and care of fall-injury related complications to prevent costly hospital readmissions.

Our study has a few limitations due to data constraints. First, the reliability and validity of self- or family member-reported fall injury and healthcare utilization data were not ascertained. Second, because the reference period was 91 days, many older adults who had injurious falls prior to the reference period were not included and annual fall rates could not be calculated. However, using this relatively short reference may mean that reports of fall injuries and related healthcare service were more accurate than if the period had been longer as older adults tend to underreport their falls. A study using 24-month self-report recall data found that 72% of individuals who received Medicare-reimbursed health care for fall-related injuries failed to self-report a fall injury when asked about it [32]. Third, sensory and memory deficits and mental health problems were based on the family respondent’s report/perceptions rather than validated scales. Fourth, the cross-sectional data used in our study can only be used to assess correlation, not causation.

## Conclusions

High ED visit and hospitalization rates among older fall victims show the personal and financial tolls falls take in late life. In addition to treating underlying causes of falls, access to effective fall prevention programs that target risk factors specific to serious injuries requiring these costly forms of care are needed. Primary care providers, EMS providers, ED staff, and other healthcare and social service provider should be trained to provide fall risk assessment and prevention. Older adults at risk of falling need fall prevention programs that can teach them specific everyday strategies and behavioral modifications to improve mobility and stability and reduce fall risks.

## Data Availability

The National Health Interview Survey data sets that were used and analyzed in the current study are available for public use: https://www.cdc.gov/nchs/nhis/index.htm.

## Abbreviations

ADL: Activities of daily living
ANOVA: Analysis of variance
CDC: The United States Centers for Disease Control and Prevention
CI: Confidence intervals
ED: Emergency department
EMS: Emergency Medical Service
NHIS: The United States National Health Interview Survey
RRR: Relative risk ratio
SE: Standard error
